# Editorial: Advances of radiomics and artificial intelligence in the management of patients with central nervous system tumors

**DOI:** 10.3389/fonc.2023.1081301

**Published:** 2023-01-19

**Authors:** Ziyan Chen, Helen Zhang, Paul J. Z. Zhang, Harrison X. Bai, Xuejun Li

**Affiliations:** ^1^ Department of Neurosurgery, Xinagya hospital, Central South University, Changsha, China; ^2^ Hunan International Scientific and Technological Cooperation Base of Brain Tumor Research, Xiangya Hospital, Central South University, Changsha, China; ^3^ Department of Diagnostic Imaging, Rhode Island Hospital and Alpert Medical School of Brown University, Providence, RI, United States; ^4^ Department of Pathology and Laboratory Medicine, Perelman School of Medicine at the University of Pennsylvania, Philadelphia, PA, United States; ^5^ Department of Radiology and Radiological Science, Johns Hopkins University School of Medicine, Baltimore, MD, United States

**Keywords:** central nervous system, radiomics, artificial intelligence, imaging, management

Central nervous system (CNS) tumors can cause severe morbidity and mortality across all populations. The average annual age-adjusted incidence rate of all CNS tumors was 24.25 per 100,000 in the US from 2014-2018 and 5.57 per 100,000 in China in 2016 (1, 2). Although rare, CNS tumors are considered as a significant contributor to cancer mortality due to their high mortality rate (3). In the US, only one-third of patients diagnosed with a malignant CNS tumor survived at least 5 years past diagnosis (4). CNS tumors are extremely heterogeneous with over 100 histological subtypes, requiring individualized management strategies (5, 6). Nowadays, there is a plethora of imaging, biological, and clinical data that can inform identification and management of patients with CNS tumors. Recent approaches allow us to extract hidden information from this data to explore the deep nature of tumors.

Radiomics has shown the capability to extract quantitative information from medical images and demonstrated robust performance in clinical diagnosis and grading, prognosis prediction and molecular parameter recognition 7). Artificial intelligence (AI) has demonstrated the potential to increase the workflow and performance of radiomics models (8). In previous studies, we implemented deep learning models to determine IDH status of glioma patients (9) and automatically segment abnormal contrast enhancement of gliomas on post-treatment magnetic resonance imaging (MRI) (10). We also reported the use of deep learning models to differentiate between glioma and germinoma (11), and meningioma and hemangiopericytoma (12), providing valuable clinical information for surgery and other treatments.

This Research Topic was introduced to highlight the use of AI-based radiomics and radiogenomics methods to improve diagnostic approaches and therapeutic options for CNS tumor patients. The papers within this Research Topic cover various types of CNS tumors including glioma, craniopharyngioma, meningioma, brain metastasis, pituitary macroadenoma etc. The research objectives are diverse and include tumor status evaluation, differential diagnosis, survival and recurrence assessment, and genomic features prediction.

Gliomas are the most common type of primary CNS tumor (13). Molecular biomarkers have been widely used in glioma tumor taxonomy and survival assessment (14). Fan et al. identified five valuable radiomics features with significant difference in WHO grade II glioma patients with different 1p/19q status using elastic net and support vector machine (SVM) algorithms. Similarly, Fang et al. established a radiomics-based machine-learning algorithm highlighting 12 radiomics features capable of predicting telomerase reverse transcriptase promoter (pTERT) mutations in patients with WHO grade II gliomas. Both studies obtained relatively high predictive performance with area under the curve (AUC) of 0.808 and 0.844, respectively. Thus, radiomics offers a promising approach to predict molecular biomarkers using radiological images, and has the potential to save patients from invasive biopsies. Qi et al. studied 223 glioma samples from The Cancer Imaging Archive (TCIA) and proposed a Voxel-based lesion-symptom mapping (VLSM) approach to detect glioblastoma (GBM) topography of proneural and mesenchymal subtypes. They found a difference in survival characteristics and proneural-mesenchymal transition (PMT) progression of samples in and outside of the VLSM-determined area. This study provides a valuable VLSM-determined area related to prognosis and PMT progression. To improve differential diagnosis, Zhang et al. trained a deep learning model to classify MRI lesions as GBM, primary central nervous system lymphoma (PCNSL), or tumefactive demyelinating lesion (TDL). The model achieved improved diagnostic performance over neuroradiologists. Imaging data aside, radiology reports remain the major point of reference for many diagnostic and treatment decisions. Cao et al. applied natural language processing to automatically interpret glioma pre-operative MRI reports and predict IDH mutation. They identified 30 glioma enhancement descriptions and established a high-performance model based on report features and age, achieving an AUC of 0.89 in predicting IDH mutation status. These varying approaches demonstrate the potential integration of a wide range of data into glioma assessment.

Meningiomas are thought to originate from arachnoid cells and account for over 30% of all primary brain tumors (15). Surgical resection is generally the preferred treatment for meningiomas. Malignant meningiomas, however, are more aggressive and have a higher risk of recurrence after surgery compared to benign meningiomas. Ko et al. applied pre-operative radiomic features evaluated by SVM to predict progression/recurrence in 128 meningioma patients. Using SVM scores, an AUC of 0.80 was achieved, with an optimal cutoff value of 0.224. Xiao et al. explored the use of 3D radiomics features extracted from multi-parameter MRI to evaluate postoperative cerebral edema exacerbation in patients with meningioma. A combined model was constructed from the established radiomics signature and clinical data. This combined model demonstrated a high prediction accuracy, with an AUC of 0.91 in the primary cohort and 0.83 in the validation cohort. This work demonstrates the potential contributions of AI-based radiomics to characterization and clinical management of meningiomas.

Brain metastases (BM) are a common manifestation of cancer that can worsen clinical outcomes (16). An estimated 20% of all patients with cancer will develop brain metastases, with most brain metastases occurring in those with lung cancer, especially non-small cell lung cancer (NSCLC) (17). Advances in neuroimaging and computer-assisted approaches have provided additional opportunities to accurately screen and precisely target intracranial lesions noninvasively. To predict survival of patients with BM from NSCLC, Chen et al. retrospectively identified 110 patients with BM from EGFR, ALK, and/or KRAS mutation-positive NSCLC and constructed a model using MRI radiomics and clinical features. The model yielded an AUC of 0.977, 0.905, and 0.947 for the EGFR, ALK, and KRAS mutation-positive groups, respectively. Zhang et al. implemented a CT-based radiomics nomogram model to predict prognosis of patients with BM from NSCLC treated with whole brain radiotherapy. Five radiomics features were selected through LASSO Cox regression and used to construct the nomogram. The resulting AUCs were 0.786 and 0.788 for short-term and long-term survival predictions, respectively. These studies demonstrate the applicability of radiomics and AI-based approaches to outcome prediction in patients with brain metastases.

Lastly, we considered studies that applied radiomics methods to biological status prediction of other tumors in CNS. Ma et al. reported the construction of a radiomics-clinical nomogram for individualized preoperative prediction of the invasiveness of adamantinomatous craniopharyngioma (ACP) before surgery. A total of 355 patients were enrolled in the study and 11 features associated with the invasiveness of ACPs were selected. A nomogram incorporating peritumoral edema and the radiomics signature reached an AUC of 0.735 in the validation cohort. This study described a potential and reliable tool for clinical decision making. Zhang et al. constructed a radiomics model based on routine MRI to predict progression/recurrence of non-functioning pituitary macroadenomas (NFPAs) after surgical resection. They used an SVM classifier to evaluate the importance of extracted parameters to build a prediction model. The model showed an accuracy of 82% and AUC of 0.78 in differentiating between progression/recurrence and non-progression/recurrence in NFPAs.

## Conclusion and perspective

The 11 papers collected in this Research Topic produced promising results in their application of radiomics and AI to the management of patients with various CNS tumors. With the rapid advancement of AI and medical imaging techniques, radiomics is assuming a larger role in cancer diagnosis and treatment, as well as biological investigation. We are encouraged by the great support from the research community for this topic; a total of 99 authors contributed to the 11 selected papers, which have already generated significant attention within the field. Nevertheless, more work is needed to advance computational precision medicine. Models with higher accuracy and robustness as well as clinical interpretability require further exploration. All studies within this Research Topic are retrospective and may be limited by smaller sample sizes. To facilitate clinical translation, additional studies may need to focus on image and data standardization between different institutions, data sharing, and prospective research. However, the present contributions of radiomics and AI-based approaches to tumor diagnosis and management indicate significant promise for the near future.

## Author contributions

All authors listed have made a substantial, direct and intellectual contribution to the work, and approved it for publication.
